# Clinical predictors of antipsychotic use in children and adolescents with autism spectrum disorders: a historical open cohort study using electronic health records

**DOI:** 10.1007/s00787-015-0780-7

**Published:** 2015-10-15

**Authors:** Johnny Downs, Matthew Hotopf, Tamsin Ford, Emily Simonoff, Richard G. Jackson, Hitesh Shetty, Robert Stewart, Richard D. Hayes

**Affiliations:** Institute of Psychiatry, Psychology and Neuroscience, King’s College London, De Crespigny Park, Box 63, SE5 8AF London, UK; South London and Maudsley NHS Foundation Trust, London, UK; University of Exeter Medical School, Exeter, UK

**Keywords:** Child and adolescence, Autism spectrum disorders, Antipsychotic medications, Psychiatric comorbidity, Challenging behaviours

## Abstract

Children with autism spectrum disorders (ASD) are more likely to receive antipsychotics than any other psychopharmacological medication, yet the psychiatric disorders and symptoms associated with treatment are unclear. We aimed to determine the predictors of antipsychotic use in children with ASD receiving psychiatric care. The sample consisted of 3482 children aged 3–17 with an ICD-10 diagnosis of ASD referred to mental health services between 2008 and 2013. Antipsychotic use outcome, comorbid diagnoses, and other clinical covariates, including challenging behaviours were extracted from anonymised patient records. Of the 3482 children (79 % male) with ASD, 348 (10 %) received antipsychotic medication. The fully adjusted model indicated that comorbid diagnoses including hyperkinetic (OR 1.44, 95 %CI 1.01–2.06), psychotic (5.71, 3.3–10.6), depressive (2.36, 1.37–4.09), obsessive–compulsive (2.31, 1.16–4.61) and tic disorders (2.76, 1.09–6.95) were associated with antipsychotic use. In addition, clinician-rated levels of aggression, self-injurious behaviours, reduced adaptive function, and overall parental concern for their child’s presenting symptoms were significant risk factors for later antipsychotic use. In ASD, a number of comorbid psychiatric disorders are independent predictors for antipsychotic treatment, even after adjustment for familial, socio-demographic and individual factors. As current trial evidence excludes children with comorbidity, more pragmatic randomised controlled trials with long-term drug monitoring are needed.

**Electronic supplementary material:**

The online version of this article (doi:10.1007/s00787-015-0780-7) contains supplementary material, which is available to authorized users.

## Introduction

Antipsychotics are the most common psychotropic medication prescribed to children with autism spectrum disorders (ASD) [1]. US-based studies suggest between 20 and 34 % of children with ASD receive antipsychotics [2, 3]. Rates are lower in Europe, between 7 and 11 % [4, 5], but appear to be increasing [6]. Two atypical antipsychotics in particular are most commonly used, risperidone and aripiprazole, which have been demonstrated to be effective in reducing “irritability” in children with ASD, but show limited impact on the core features of ASD [7].

Clinicians and families face a difficult task when deciding whether antipsychotic treatment is indicated. Evidence from antipsychotic trials in childhood ASD is derived from samples that bear little resemblance to children typically seen in clinical practice, as they exclude children with formally diagnosed psychiatric comorbidity [8]. Another problem is the almost exclusive focus of trials on irritability as a target symptom in ASD. Irritability is a highly prevalent symptom in clinical settings, it has no standard taxonomy, and is associated with most childhood mental health problems [9]. Therefore, based on trial evidence, the type and severity of childhood ASD-related irritability symptoms, which warrant antipsychotic treatment, are unclear. Furthermore, antipsychotic medication does not have UK marketing authorisation for use in childhood ASD, although risperidone is licensed for use in the short-term management of aggression in children with conduct disorder [10]. Balancing antipsychotic risk-benefit profiles is further complicated by little safety evidence being available for children with ASD [11, 12]. Antipsychotic use for children in general is associated with a number of adverse health outcomes, most commonly extrapyramidal side effects, obesity and hyperprolactinaemia [13]. Given the limited evidence base, NICE guidelines advocate cautious antipsychotic prescribing in children with ASD and only to treat severe challenging behaviours (also known as ‘behaviours that challenge’) such as aggression, self-injury and impulsive/dangerous behaviours [14].

It remains unclear how current evidence, licensing and guidance for antipsychotic use in children with ASD are applied clinically [15]. There are very few UK-based naturalistic studies of prescribing in children with ASD, and, as yet, no examinations of the diagnostic predictors of antipsychotic use [4]. Comorbid psychiatric disorders are common (and frequently multiple) in children with autism spectrum disorders and may be targets for intervention [16]. Current knowledge is largely based on parent reports in US surveys which indicate that antipsychotics are used predominantly to treat comorbid diagnoses (e.g. depression, bipolar, anxiety, conduct disorder and attention deficit hyperactivity disorders) in children with ASD [2, 3]. However, these findings may not generalise to non-US clinical populations as US antipsychotic marketing [17], prescribing policy [18, 19] and practice differ markedly to the other Western Countries [1, 20]. Given that the majority of the aforementioned studies report cross-sectional findings from retrospective parental accounts of both comorbidity and past medication use, the direction of effect is unclear, and recall bias may obscure true prescribing patterns. Furthermore, these studies do not account for important confounding factors, such as psychosis, adaptive function, and intellectual disability that may lead to an overestimate of the association between certain comorbidities and antipsychotic use.

To clarify how antipsychotics are used in childhood ASD, we explored the clinical factors that predicted antipsychotic prescribing. We conducted a historical cohort study using the anonymised electronic health records of children with ASD treated by UK child and adolescent mental health services (CAMHS). As challenging behaviours (or ‘behaviours that challenge’) are symptoms that cut across most childhood psychopathology, we hypothesised that the common psychopathologies are comorbid with ASD including hyperkinetic, oppositional and conduct, depression and anxiety disorders would all show longitudinal associations with antipsychotic use. We also examined whether associations between comorbidity and antipsychotics were attenuated after we controlled for challenging behaviours, given that these are the most common non-psychotic symptoms formally recognised as targets for antipsychotic treatment by current national ASD management guidelines [14].

## Methods

### Study setting

This study used data extracted from the anonymised, electronic clinical records of children referred to South London and Maudsley NHS Foundation Trust (SLaM) between 1 January 2008 and 31 December 2013. Over this period, SLaM provided all aspects of specialist mental healthcare to a catchment population of approximately 300,000 children resident within four London boroughs (Lambeth, Southwark, Lewisham, Croydon). In addition to the district services, SLaM provided specialist inpatient and outpatient ASD assessment and treatment services for young people from across the UK. Each borough had a dedicated multidisciplinary service for children, which accepted referrals for school age children (4–18 years; exceptionally cases are accepted below this age) with suspected or previously confirmed ASD, displaying emotional or behavioural difficulties. Children were referred from primary care, child health, and educational and social care services, and typically underwent a multidisciplinary assessment by CAMHS clinicians. Primary and secondary psychiatric disorders were diagnosed by CAMHS using the ICD-10 multi-axial classification system [21]. Semi-structured validated assessments, for example, the Autism Diagnostic Observation Schedule (ADOS) [22] were used if an ASD diagnosis was unclear after initial assessment (the SLaM autism pathway is available from the author on request). Compared with expert consensus, there is a high specificity for ASD diagnoses by clinicians working at a district level [23]. Socio-demographic characteristics and clinical information were recorded using computerised assessment proforma, which included the Strengths and Difficulties Questionnaire (SDQ) [24].

The Clinical Record Interactive Search (CRIS) system was used to provide an anonymised, electronic mental health records database to search on structured data and free text fields on over 34,400 child and adolescent cases referred to SLaM services. Generalised Architecture for Text Engineering (GATE), a natural language processing architecture was applied to extract data from the free text (progress notes, correspondence, etc.) and structured fields [25].

### Study sample

Cases were part of an open clinical cohort (entering and leaving the study at different time points) and included children aged 3–17 years with a diagnosis of ASD (International Classification of Diseases, 10th Revision (ICD-10) F84.0, F84.1, F84.5, F84.9) [21] recorded between 1 January 2008 and 31 December 2013. Children were excluded if any past course of antipsychotic treatment was noted in the clinical record in the year prior to the observation period.

### Measurements

#### Outcome: antipsychotic use

Antipsychotic use outcome data were extracted from free text fields held in the SLaM Case Register, the SLaM pharmacy dispensing database and structured medication fields in the electronic health record using validated GATE software extraction methods [26]. Antipsychotic use was measured during the observation period (01/01/2008–31/12/2013). The start date for antipsychotic use was recorded and categorised as a present/absent outcome.

#### Exposure: psychiatric comorbidity and intellectual disability

The main exposure was ICD-10 recorded comorbid psychiatric diagnoses, which were extracted from free text and structured fields. ICD-10 Axis one comorbid diagnoses were categorised into: psychotic (F1x.5, F20–F29, F31, F32.3, F33.3), depressive disorders (F32), anxiety, stress and emotional (F40–41, F43–F48, F93), obsessive–compulsive (F42), hyperkinetic (F90), oppositional defiant and conduct (F91–F92) and tic (F95). Low frequency psychiatric diagnoses were collapsed into a single category labelled “Other”. In addition, children were categorised according to presence of an ICD-10 Axis three diagnosis of intellectual disability (F70–F79).

Prescribing decisions were recorded contemporaneously, but there was often a short administrative lag before diagnostic reports appeared in the electronic medical record. These reports contained detailed clinical assessments conducted during the pre-medicated period. To permit inclusion of these longitudinally collected clinical data, but also ensure comorbidity exposures occurred prior to antipsychotic use, comorbid diagnoses were only coded as present if they were recorded before, or up to 30 days after, recording of antipsychotic medication.

#### Covariates

All covariates were extracted from the medical record during the initial CAMHS assessment period, and prior to antipsychotic use. Measures of challenging behaviours were taken from the SLaM risk assessment proforma. We chose assessment items with high face validity for challenging behaviours, as described by expert consensus in national guidance [14], including physical aggression against self (self-injury), violence and aggression to others or property (aggression), harm through loss of self care such as not drinking or eating (self-neglect), impulsive and dangerous acts (high-risk behaviours), and habitual behaviours related to intellectual disability such as rocking or skin picking that can cause injury (ID-related harm). Clinicians rated severity along a 4-point categorical scale: ‘None’, ‘Low’, ‘Moderate’, or ‘High’ (available on request). For ease of clinical interpretation and to ensure adequate numbers in each category, this scale was converted into a binary variable for each behaviour domain (Moderate or High rating categorised as High risk = 1; None or Low ratings, low risk = 0). Children’s adaptive functioning was rated using Children’s Global Assessment Scale (CGAS) [27], except for those children with significant ID, where the Developmental Disabilities CGAS was used in some cases [28]. Higher scores (range 0–100) are associated with better functioning.

Demographic and family covariates consisted of gender, ethnicity, history of parental mental illness, and clinician-rated levels of parental concern for their child’s symptoms at their initial presentation to CAMHS, which were retrieved using CRIS from structured fields in the source dataset. Age at CAMHS assessment was calculated at the date of the first recorded diagnosis of autism spectrum disorder within the clinical record. UK Census data provided small area (average 400 households) level deprivation scores [29].

Emotional, hyperactive and conduct problem domains were assessed via the caregiver versions of the 25-item Strengths and Difficulties Questionnaire which has sound psychometric properties in clinical samples [30]. These were available in the clinical record for a third of the sample (*n* = 1234, 35 %).

### Analysis

To authenticate clinically recorded common comorbid conditions (depression and anxiety, hyperkinetic and conduct disorders), we used a sub-group of the cohort with completed SDQs (*n* = 1234). Independent sample *t* tests were used to test for statistical differences in parental reported SDQ psychopathology subscale scores (emotional, hyperactivity, conduct problem subscales) for children with and without these common comorbid conditions.

Logistic regression was used to examine whether antipsychotic use was predicted by demographic characteristics, psychiatric comorbidities, intellectual disability, adaptive function, behaviours that challenge, parental characteristics and neighbourhood deprivation. Multivariable analysis was then conducted to examine the effect of each of these variables on antipsychotic use after adjusting all other individual and contextual covariates (listed in Table 1). The following sensitivity analyses were carried out: (1) using non-aggregated challenging behaviour categories (4 levels) as the binary variable may have introduced residual confounding; (2) excluding those who came from outside the local catchment (these individuals may have had substantial contact with non-SLaM services not represented in the CRIS source dataset); (3) selecting those with only one comorbid disorder and modelling the effect of a single comorbidity on antipsychotic use (without adjusting for the full set of covariates). All analyses were conducted using Stata version 12.

**Table 1:** Individual and contextual characteristics of 3482 children with autism spectrum disorders and antipsychotic use referred to local and specialist child and adolescent mental health services

Child age category	At CAMHS assessment		At antipsychotic use	
	*n* (%)	Mean (SD)	*n* (%)	Mean (SD)
Early (3–6 years)	362 (10.4 %)	4.9 (0.77)	3 (0.9 %)	5.7 (0.3)
Mid (6–12 years)	1664 (47.8 %)	9.0 (1.69)	89 (25.6 %)	9.6 (1.4)
Late (13–17 years)	1456 (41.8 %)	14.8 (1.66)	256 (73.5 %)	15.1 (1.6)

	Total sample *n* = 3482		Sample receiving antipsychotics *n* = 348	
Male	2686 (77.1 %)		249 (71.6 %)	
Female	796 (22.9 %)		99 (28.4 %)	
Ethnicity				
White British	1683 (48.3 %)		208 (59.8 %)	
White other	170 (4.9 %)		11 (3.2 %)	
East Asian	68 (2.0 %)		9 (2.6 %)	
British/Black African	651 (18.7 %)		57 (16.4 %)	
British/Black Caribbean	130 (3.7 %)		5 (1.4 %)	
Mixed Heritage	386 (11.1 %)		37 (10.6 %)	
South Asian	84 (2.4 %)		11 (3.2 %)	
Not stated	310 (8.9 %)		10 (2.8 %)	
Adaptive function				
Children’s Global Assessment Scale (CGAS)^a^				
0–25 (most impaired)	174 (5.7 %)		50 (15.0 %)	
25–50	1346 (43.8 %)		219 (65.6 %)	
50–75	1465 (47.7 %)		62 (18.5 %)	
75–100	89 (2.90 %)		3 (0.9 %)	
Challenging behaviours self injury^b^				
Low	2368 (83.3 %)		171 (57.0 %)	
High	474 (16.7 %)		129 (43.0 %)	
ID-related harm^c^				
Low	1600 (59.3 %)		142 (48.6 %)	
High	1097 (40.7 %)		150 (51.4 %)	
Aggression^d^				
Low	1724 (59.7 %)		100 (32.3 %)	
High	1165 (40.3 %)		210 (67.7 %)	
Self-neglect^e^				
Low	2562 (89.5 %)		231 (75.0 %)	
High	300 (10.5 %)		77 (25.0 %)	
High-risk behaviours^f^				
Low	2175 (76.9 %)		161 (53.5 %)	
High	654 (23.1 %)		140 (46.5 %)	
Family characteristics				
Caregiver mental illness^g^				
No	2309 (77.1 %)		247 (77.2 %)	
Yes	685 (22.9 %)		73 (22.8 %)	
Caregiver substance misuse^g^				
No	2793 (93.3 %)		304 (95.0 %)	
Yes	201 (6.7 %)		16 (5.0 %)	
Parental concern^h^				
Low	876 (30.1 %)		33 (10.6 %)	
High	2033 (69.9 %)		278 (89.4 %)	
Neighbourhood characteristics^i^				
Level of deprivation (tertiles)				
1st (least deprived)	1064 (32.8 %)		142 (44.4 %)	
2nd	1093 (33.7 %)		95 (29.7 %)	
3rd (most deprived)	1089 (33.6 %)		83 (25.9 %)	

Missing cases = ^a^408, ^b^640, ^c^785, ^d^593, ^e^ 620, ^f^653, ^g^488, ^h^ 573, ^i^236

## Results

Over the 6-year observation period, we identified 3482 children aged below 18 years (2686 male and 796 female) with a diagnosis of ASD. The mean (SD) exposure to child mental health services, defined as the time between the date of recorded ASD diagnosis and the end of the observation period or date of 18th Birthday (whichever sooner) was 968 (597) days. Three hundred and forty-eight children were prescribed antipsychotics, mainly risperidone (55 %, *n* = 191) and aripiprazole (32 %, *n* = 112). All were receiving adjunctive psycho-social interventions. Table 1 shows the characteristics of the total sample and those prescribed antipsychotics. Nearly 75 % of children prescribed antipsychotics were in the adolescent age range (age 13–18 years), representing a sixfold risk (OR 6.29, 95 % CI 3.40–12.1) relative to early childhood (age 3–6 years).

In our authentication analyses, we found that ASD children diagnosed with comorbid emotional (depression and anxiety), hyperkinetic or conduct disorders had significantly higher SDQ subscales scores within their respective SDQ domains (emotional, hyperactive, conduct) compared with children without the respective comorbid diagnosis (see supplementary Table 1). ICD-10 recorded comorbid psychiatric diagnoses were present in 54 % of the sample, a quarter diagnosed with a hyperkinetic disorder, and 20 % diagnosed with intellectual disability. Table 2 provides further details of comorbid psychiatric diagnoses by antipsychotic use. Antipsychotics were prescribed to approximately half of children with ASD and psychotic disorder, and over a quarter of children diagnosed with obsessive–compulsive disorder, or tic disorders.

**Table 2:** Prevalence of comorbid psychiatric disorder and antipsychotic treatment in 3482 children with autism spectrum disorders

	Total sample *n* = 3482	Sample receiving antipsychotics *n* = 348
Any comorbid disorder		
−	1585 (45.5 %)	63 (18.1 %)
+	1897 (54.5 %)	285 (81.9 %)
Hyperkinetic		
−	2620 (75.2 %)	227 (65.2 %)
+	862 (24.8 %)	121 (34.8 %)
Oppositional and conduct		
−	3226 (92.7 %)	297 (85.3 %)
+	256 (7.3 %)	51 (14.7 %)
Depression		
−	3328 (95.6 %)	312 (89.7 %)
+	154 (4.4 %)	36 (10.3 %)
Anxiety, emotional and stress		
−	3203 (92.0 %)	303 (87.1 %)
+	279 (8.0 %)	45 (12.9 %)
Obsessive–compulsive		
−	3385 (97.2 %)	322 (92.5 %)
+	97 (2.8 %)	26 (7.5 %)
Tic		
−	3431 (98.5 %)	335 (96.3 %)
+	51 (1.5 %)	13 (3.7 %)
Psychosis		
−	3366 (96.8 %)	294 (84.5 %)
+	116 (3.3 %)	54 (15.5 %)
Intellectual disability		
−	2826 (81.2 %)	234 (67.2 %)
+	656 (18.8 %)	114 (32.8 %)
Other**		
−	3353 (96.3 %)	330 (94.8 %)
+	129 (3.7 %)	18 (5.2 %)

(−) comorbidity absent (+) comorbidity present

**Remaining, rarely occurring diagnoses, were collapsed into a single category labelled Other (includes ICD-10 F50 eating disorders, F04–09 organic disorders, F1x.1–4 substance misuse, F94.1–2 attachment disorders)

In the adjusted model, positive associations with antipsychotic use remained significant for age at the time of assessment, clinician-rated aggression, self-injurious behaviour, and high parental concern for their child’s symptoms at initial presentation (see Table 3). In addition, adaptive function and the presence of caregiver substance misuse showed strong inverse associations with antipsychotic use. Associations with ethnicity, caregiver mental illness and neighbourhood deprivation were non-significant. Table 4 shows that a number of comorbid ICD-10 mental disorders, even after adjustment for all other covariates and comorbidities, remained significantly associated with antipsychotic use including hyperkinetic (OR 1.44, 95 %CI 1.01–2.06), psychotic (OR 5.71, 3.3–10.6), depressive (2.36, 1.37–4.09), obsessive–compulsive (2.31, 1.16–4.61) and tic disorders (2.76, 1.09–6.95). These associations remained when antipsychotic use was compared between ASD children with no-comorbidity to those who had the specific comorbidity alone, rather than multiple comorbidities (for example, only comorbid hyperkinetic disorder, see supplementary Table 2).

**Table 3:** Multivariable model of antipsychotic use in children with ASD by socio-demographic characteristics and other covariates

	OR (95 % CI) (*n* = 3482)	*P*	aOR (95 % CI)^a^	*P*
Female sex (vs male)	1.39 (1.09–1.79)	0.009	1.02 (0.71–1.46)	0.89
Age at CAMHS assessment	1.18 (1.15–1.23)	<0.0001	1.11 (1.05–1.16)	<0.001
Ethnicity				
White British	Reference			
White other	0.49 (0.26–0.92)	0.026	0.62 (0.24–1.55)	0.31
East Asian	1.08 (0.52–2.21)	0.83	0.91 (0.35–2.31)	0.84
British/Black African	0.68 (0.50–0.92)	0.014	1.14 (0.73–1.78)	0.55
British/Black Caribbean	0.28 (0.11–0.70)	0.006	0.56 (0.21–1.54)	0.26
Mixed Heritage	0.75 (0.52–1.09)	0.13	0.78 (0.46–1.32)	0.36
South Asian	1.06 (0.56–2.05)	0.84	1.26 (0.47–3.32)	0.70
Not stated	0.24 (0.12–0.45)	<0.001	0.27 (0.09–0.80)	0.02
Adaptive function				
Children’s Global Assessment Scale (CGAS)	0.95 (0.94–0.95)	<0.0001	0.96 (0.95–0.97)	<0.0001
Challenging behaviours				
Self-injury	4.80 (3.72–6.20)	<0.0001	1.85 (1.30–2.63)	<0.0001
ID-related harm	1.63 (1.27–2.07)	<0.0001	0.72 (0.49–1.06)	0.10
Aggression	3.57 (2.77–4.59)	<0.0001	2.14 (1.50–2.06)	<0.0001
Self-neglect	3.48 (2.61–4.67)	<0.0001	1.20 (0.78–1.80)	0.35
High-risk behaviours	3.40 (2.66–4.35)	<0.0001	1.22 (0.86–1.73)	0.27
Family characteristics				
Caregiver mental illness	1.0 (0.75–1.31)	0.98	0.87 (0.60–1.26)	0.47
Caregiver substance misuse	0.71 (0.42–1.20)	0.20	0.57 (0.30–1.08)	0.09
High parental concern	4.05 (2.79–5.85)	<0.0001	2.02 (1.27–3.22)	0.003
Neighbourhood				
Level of deprivation (tertiles)				
1st (least deprived)	Reference			
2nd	0.62 (0.47–0.81)	0.001	0.82 (0.56–1.19)	0.31
3rd (most deprived)	0.54 (0.40–0.71)	<0.0001	0.91 (0.62–1.35)	0.65

*OR* odds ratio, *CI* confidence intervals

^a^aOR, adjusted Odds Ratio, fully adjusting for all other covariates within the table, i.e. socio-demographic (age at CAMHS assessment, gender and ethnicity) and parental and neighbourhood characteristics, challenging behaviours, adaptive function and co-existing ICD-10 mental and behavioural disorder groupings: hyperkinetic (F90), depressive disorders (F32), psychosis (F1x.5, F20–F29, F31, F32.3, F33.3) oppositional and conduct (F91–F92), anxiety, stress and emotional (F40–41, F43–F48, F93), obsessive–compulsive (OCD, F42), tic (F95), intellectual disability (ID, F70–F79) and other psychiatric diagnosis

**Table 4:** Multivariable model of antipsychotic use in a cohort of children with ASD by psychiatric comorbidity

	OR (95 % CI) (*n* = 3482)	*P*	aOR^a^ (95 % CI)	*P*
Any comorbid disorder	4.27 (3.22–5.66)	<0.0001	–	–
Hyperkinetic	1.73 (1.36–2.18)	<0.0001	1.44 (1.01–2.06)	0.042
Oppositional and conduct	2.47 (1.77–3.43)	<0.0001	1.55 (0.96–2.51)	0.073
Depression	2.95 (1.99–4.36)	<0.0001	2.36 (1.37–4.09)	0.002
Anxiety, emotional and stress	1.84 (1.31–2.59)	<0.0001	1.20 (0.72–1.98)	0.484
Obsessive–compulsive	3.48 (2.19–5.53)	<0.0001	2.31 (1.16–4.61)	0.017
Tic	3.16 (1.67–5.99)	<0.0001	2.76 (1.09–6.95)	0.032
Psychosis	9.1 (6.19–13.4)	<0.0001	5.71 (3.28–10.6)	<0.0001
Intellectual disability	2.33 (1.82–2.97)	<0.0001	1.68 (1.11–2.53)	0.015
Other^b^	1.49 (0.89–2.48)	0.13	1.62 (0.83–3.16)	0.157

^a^aOR, adjusted Odds Ratio, adjusting for socio-demographic and parental and neighbourhood characteristics, challenging behaviours, adaptive function and co-existing ICD-10 Mental and behavioural disorder groupings: hyperkinetic (F90), depressive disorders (F32), psychosis (F1x.5, F20–F29, F31, F32.3, F33.3), oppositional and conduct (F91–F92), anxiety, stress and emotional (F40–41, F43–F48, F93), obsessive–compulsive (OCD, F42), tic (F95), intellectual disability (ID, F70–F79) and other psychiatric diagnosis

^b^Remaining, rarely occurring diagnoses, were collapsed into a single category labelled Other (includes ICD-10 F50 eating disorders, F04–09 organic disorders, F1x.1–4 substance misuse, F94.1–2 attachment disorders)

Specified sensitivity analyses that used non-aggregated behaviour categories produced little change to the overall pattern of results in the fully adjusted models, with the direction and magnitude of effect being consistent across the comorbidities. Similarly, removing the children resident outside the local catchment area from the sample (*n* = 1170, 33 %) produced little change, except for oppositional defiant and conduct disorder, where the sensitivity analysis produced very imprecise estimates due to the low number of children within the diagnostic category.

## Discussion

In the largest study to date using non-administrative, clinical mental health records in ASD, we found antipsychotic prescribing for children with ASD was strongly associated with comorbidity. Intellectual disability and psychiatric comorbidities, including hyperkinetic, depression, psychotic, obsessive–compulsive and tic disorders, were all associated with antipsychotic treatment, even after controlling for clinician-rated challenging behaviour symptoms at initial assessment. We also found increasing age, aggression, self-injurious behaviour, level of adaptive function, and parental concern were all significant predictors of antipsychotic use.

The observed association between antipsychotic use and age is consistent with previous ASD studies [2, 31]. Over two-thirds of children treated with antipsychotics were adolescents. This highlights the need for more trials that include this age group but also suggests that treatment acceptability, and hence trial recruitment, will be more feasible than in younger children. Social factors also appeared to play a role; clinicians who perceived greater parental concern for children’s presenting symptoms were more likely to prescribe antipsychotic treatment. We are not aware of any prior studies that measure parental influences on antipsychotic use in ASD; however, our finding is consistent with previous studies that show a positive association between parental strain and medication treatment for childhood disruptive disorders [32]. Consistent with several other investigations in clinical samples [33, 34], our unadjusted analyses suggest that there may be discrepancies between ethnic groups regarding prescribing antipsychotic medication to children. However, in keeping with a more recent study on psychotropic treatment in children, we found that after adjustment for markers of clinical severity, ethnicity was no longer significantly associated with antipsychotic use [35].

Using a historical cohort design in a clinical sample of children with ASD, this is the first longitudinal study of challenging behaviours and psychiatric comorbidity profiles as predictors of antipsychotic use. Our results suggest that clinicians are using antipsychotics where they are known to be efficacious [7]; to target aggression and self-injurious behaviours. Many studies so far have been hindered by parental report of comorbidities and medication use, retrospective or cross-sectional design, or the confounding effect of unmeasured psychiatric symptoms and disorder severity not being accounted for [2, 3, 36]. In addressing these limitations we found that, unlike a number of US studies, antipsychotics were not significantly associated with comorbid anxiety, emotional and stress disorders [2, 3, 36].

This study has a number of strengths: we used longitudinally collected clinician recorded data in an unselected population of children and adolescents with ASD referred to CAMHS to study off-label antipsychotic use. This avoids the non-response or recall bias issues that may arise in surveys of parents. Our sample included the entire psychiatric population of four south London boroughs for school age children (4–18 years) with suspected or previously confirmed ASD and displaying emotional or behavioural difficulties, in addition to children from other areas of the UK referred to National and Specialist services. However, because we studied a cohort enriched by national referrals, psychiatric comorbidity and antipsychotic treatment, prevalence should not be taken as representative rates of the children with ASD in the general population.

Our study has limitations. First, an ASD diagnosis may ‘overshadow’ other psychiatric diagnoses and reduce the likelihood of clinicians recording additional psychiatric diagnoses. For example, ICD-10 criteria preclude the diagnosis of hyperkinetic disorder being given once ASD is established, which may lead to an underestimate of the association between hyperkinetic comorbidity and antipsychotic use. That said, many clinicians override this instruction based on recent evidence from clinical and treatment studies. Second, the type of assessment and treatments offered to families may vary by clinician. In our analysis, we lacked detailed information about the assessing and prescribing clinician and could not account for variation in practice. Third, we did not include physical comorbidities (e.g. epilepsy, obesity), other pharmacological treatments or duration of psycho-social interventions which may act as potential confounders to antipsychotic use. Fourth, we did not apply a research scale to measure challenging behaviours [37]. Instead, we used assessment items commonly mandated for use in clinical mental health services [38], which could likely aid study replication in other UK settings. Fifth, we coded comorbid disorders preceding and up to 30 days post antipsychotic use, which prevented the exclusion of pre-medication diagnostic reports. Theoretically, this could introduce an observer bias, as the intensity of observation by the CAMHS service post antipsychotic treatment may increase a child’s risk of having a clinically recorded comorbid disorder. However, iatrogenic comorbid psychiatric conditions are very unlikely to develop or be recorded within this short timeframe. Last, due to limitations in the free text coding and extraction process, we cannot exclude residual confounding as an influence on our findings, especially within the broad diagnostic categories of psychotic disorder or intellectual disability. We were unable to accurately categorise the degree of intellectual disability, nor characterise the severity or duration of psychotic disorder from the electronic health records. However, we did address potential confounding due to severity of psychotic disorders and intellectual disability to some extent by the inclusion of Children’s Global Assessment Score [27] as a covariate in the final multivariable models. Residual confounding may remain nonetheless.

Our findings reflect the complexity of assessing and treating comorbid psychiatric disorders in ASD. For example, ASD and psychotic disorders pose a common diagnostic challenge to clinicians given their overlapping characteristics and high potential for co-occurrence [39, 40]. We found only 47 % of children with ASD and psychosis received antipsychotics. This low treatment rate may be due to diagnostic uncertainty. Children with ASD may be more likely to have their diagnosis of psychosis withdrawn after further clinical assessment, and before the initiation of antipsychotic treatment. A second reason may relate to clinicians, children and their families deciding that some psychotic symptoms in ASD do not warrant antipsychotic treatment. Evidence that may dissuade those from starting antipsychotic treatment includes findings from longitudinal studies, which show fluctuating psychotic symptoms in children with features of autism can have a relatively benign course [41, 42].

Our findings provide a detailed account of current antipsychotic prescribing practices in a clinical population of children with ASD, which show that aggression and self-injurious behaviours are significantly associated with antipsychotic use. Irritability may be an underlying treatment target driving the association between these behaviours and antipsychotic treatment. It may also underlie the associations we found between antipsychotic use and hyperkinetic, depressive and obsessive–compulsive disorders. Alternatively, disorder specific symptoms may be targeted. For example, trial data have shown that risperidone and aripiprazole both significantly reduce hyperactivity and obsessional compulsive symptoms in ASD [37, 43]. In addition, the study findings highlight the need for further research in childhood ASD to determine which psychotic phenomena warrant antipsychotic treatment. This would help clinicians reduce the harms associated with both antipsychotic under-use (i.e. prolonging the duration of untreated psychosis) and over-use. Future research might valuably include children without ASD as comparison groups and employ more intricate text extraction methodologies to assess symptom-specific severity and impairment. This would reduce the residual confounding effects that may occur when using broad diagnostic categories, and determine whether comorbid psychiatric diagnoses in clinical practice are approached differently in children with ASD.

Our findings highlight a mismatch between current clinical trials and the evidence needed to support clinical practice in ASD. Antipsychotic use was much greater in adolescents and for those with comorbid diagnoses. However, most published trials exclude children with comorbidity and rarely recruit adolescents [7, 8, 11]. Importantly, we show social factors play a significant part in antipsychotic use. This provides an impetus to examine the association of antipsychotic treatment against contextual, as well as clinical factors. Controversy between the potential harm of both over- and under-use of antipsychotics in children with ASD continues, and underlies considerable public concern [15]. Large-scale cohort studies in real world settings, such as ours, eventually leading to pragmatic trials using electronic patient records, will help this debate become better informed.

## Electronic supplementary material

Below is the link to the electronic supplementary material.

Supplementary material 1 (DOCX 99 kb)
