# Supplementary material for: Clinical predictors of antipsychotic use in children and adolescents with autism spectrum disorders: a historical open cohort study using electronic health records
Source: Eur Child Adolesc Psychiatry. 2015 Oct 15;25:649–58. doi: 10.1007/s00787-015-0780-7 (PMC4889626; doi:10.1007/s00787-015-0780-7)
Supplement: Supplementary file 1 — Supplementary material 1 (DOCX 99 kb) [file 787_2015_780_MOESM1_ESM.docx]

Title : Clinical predictors of antipsychotic use in children and adolescents with autism spectrum disorders: a historical open cohort study using electronic health records

Johnny Downs, Matthew Hotopf , Tamsin Ford, Emily Simonoff , Richard G. Jackson, Hitesh Shetty, Robert Stewart and Richard D. Hayes

Correspondence to: Dr Johnny Downs, Box 63, Department of Psychological Medicine, Institute of Psychiatry, King’s College London, De Crespigny Park, SE5 8AF London, UK. Phone: +44 (0)20 3228 8553 Fax: +44 (0)20 3228 8551, Email: [johnny.downs@kcl.ac.uk](mailto:johnny.downs@kcl.ac.uk).

| **Supplementary Table 1: Comorbid disorders diagnosed by clinicians and validated against parental Strength and Difficulties Questionnaire subscale score in sub-sample of children with ASD (n=1234)*** | | | | |
| --- | --- | --- | --- | --- |
| Clinical Diagnoses | | SDQ subscale (df=1234)  (mean, SD) | | |
|  |  | Emotional | Conduct | Hyperactivity |
| Depressive and Emotional Disorders  [Depressive disorders (F32), anxiety, stress and emotional (F40-41, F43-F48, F93), Obsessive-compulsive (F42)] | Present (n=171) | 6.5(2.8)^a^ | 3.72(2.5) | 6.35(2.8) |
|  | Absent(n=1063) | 4.6(2.5)^a^ | 4.25(2.5) | 7.36(2.5) |
| Externalizing Disorders  Oppositional / Conduct Disorders  (F91-F92) | Present (n=81) | 5.27(2.7) | 6.0(2.5)^b^ | 7.61(2.3) |
|  | Absent(n=1153) | 4.85(2.8) | 4.14(2.5)^b^ | 7.19(2.6) |
|  |  |  |  |  |
| ADHD (Hyperkinetic (F90) | Present (n=345) | 4.52(2.8) | 5.24(2.5) | 8.44(1.9)^c^ |
|  | Absent(n=889) | 5.02(2.8) | 3.88(2.4) | 6.74(2.7)^c^ |
| ^a^ *t* =8.57, p<0.001  ^b^ *t* = 6.5, p<0.001 ^c^ *t* =10.8, *P*<0.001  * This sub-sample (n=1234) were broadly representative with the remaining cohort. Male gender (77.9% vs 76.8%) mean age at recorded ASD diagnosis (10.5 vs 11.2 years), White British (43.5% vs 46.3%) SDQ relevant clinical diagnoses: Emotional disorder (15.1% vs 12.8%) and Hyperkinetic (23.2% vs 27.2%), Conduct (6.4% vs 7.9%); and antipsychotic use (9.4% vs 10.3%). | | | | |

| **Supplementary Table 2: A comparison of antipsychotic treatment between children with no comorbidity and singleton comorbid disorder only in Autism Spectrum Disorders.** | | | | | | | | | | | | | |
| --- | --- | --- | --- | --- | --- | --- | --- | --- | --- | --- | --- | --- | --- |
|  | |  | | No antipsychotics | | | Receiving  antipsychotics | | | O.R (95% C.I.) | | | *P* |
|  | |  | | n (%) | | | n (%) | | |  | | |  |
|  | |  | |  | | |  | | |  | | |  |
| ASD (no comorbid disorder) |  | | 1522(96.0) | | 63(4.0) | | | reference | | |  | | |
|  |  | |  | |  | | |  | | |  | | |
| **Singleton comorbid**  **disorder** | | | | |  | | |  | | |  | | |
|  | |  | |  | | |  | | |  | | |  |
| Hyperkinetic | |  | | 454(91.4) | | | 43(8.7) | | | 2.29(1.53-3.41) | | | <0.0001 |
|  | |  | |  | | |  | | |  | | |  |
|  | |  | |  | | |  | | |  | | |  |
| Oppositional and Conduct | |  | | 73(93.6) | | | 5(6.4) | | | 1.65(0.64-4.23) | | | 0.29 |
|  | |  | |  | | |  | | |  | | |  |
|  | |  | |  | | |  | | |  | | |  |
| Depression | |  | | 59(81.9) | | | 13(18.1) | | | 5.32 (2.77-10.2) | | | <0.0001 |
|  | |  | |  | | |  | | |  | | |  |
| Anxiety, Emotional and Stress | |  | |  | | |  | | |  | | |  |
|  |  |  | | 136(95.1) | | | 7(4.9) | | | 1.23(0.55-2.77) | | | 0.59 |
|  |  |  | |  | | |  | | |  | | |  |
|  | |  | |  | | |  | | |  | | |  |
| Obsessive Compulsive | |  | | 51(82.3) | | | 11(17.7) | | | 5.21 (2.59-10.5) | | | <0.0001 |
|  | |  | |  | | |  | | |  | | |  |
|  | |  | |  | | |  | | |  | | |  |
| Tic | |  | | 20(86.9) | | | 3(13.0) | | | 3.61(1.04-12.5) | | | 0.03 |
|  | |  | |  | | |  | | |  | | |  |
|  | |  | |  | | |  | | |  | | |  |
| Psychosis | |  | | 17(42.5) | | | 23(57.5) | | | 32.7(16.6-64.2) | | | <0.0001 |
|  | |  | |  | | |  | | |  | | |  |
|  | |  | |  | | |  | | |  | | |  |
| Intellectual Disability | |  | | 329(85.7) | | | 55(14.3) | | | 4.04 (2.75-5.91) | | | <0.0001 |
|  | |  | |  | | |  | | |  | | |  |
|  | |  | |  | | |  | | |  | | |  |
| Other ** | |  | | 43(95.6) | | | 2(4.4) | | | 1.12(0.27-4.74) | | | 0.87 |
|  | |  | |  | |  | | |  | | |  | |
| ** includes eating disorders, organic disorders, substance misuse, attachment disorders, OR, odds ratio. CI Confidence Intervals | | | | | | | | | | |  | | |
